# Male 11β-HSD1 Knockout Mice Fed Trans-Fats and Fructose Are Not Protected From Metabolic Syndrome or Nonalcoholic Fatty Liver Disease

**DOI:** 10.1210/en.2016-1357

**Published:** 2016-07-12

**Authors:** Dean P. Larner, Stuart A. Morgan, Laura L. Gathercole, Craig L. Doig, Phil Guest, Christopher Weston, Jon Hazeldine, Jeremy W. Tomlinson, Paul M. Stewart, Gareth G. Lavery

**Affiliations:** Institute of Metabolism and Systems Research (D.P.L., S.A.M., C.L.D., P.G., G.G.L.), University of Birmingham, Birmingham B15 2TT, United Kingdom; Centre for Endocrinology, Diabetes and Metabolism (D.P.L., S.A.M., C.L.D., P.G., G.G.L.), Birmingham Health Partners, Birmingham B15 2TH, United Kingdom; Oxford Centre for Diabetes Endocrinology and Metabolism (L.L.G., J.W.T.), University of Oxford, Churchill Hospital, Headington, Oxford OX3 7LJ, United Kingdom; Institute for Immunology and Immunotherapy (C.W.), University of Birmingham, Birmingham B15 2TT, United Kingdom; Institute of Inflammation and Ageing (J.H.), University of Birmingham, Birmingham B15 2TT, United Kingdom; and Faculty of Medicine and Health (P.M.S.), University of Leeds, Leeds LS2 9JT, United Kingdom

## Abstract

Nonalcoholic fatty liver disease (NAFLD) defines a spectrum of conditions from simple steatosis to nonalcoholic steatohepatitis (NASH) and cirrhosis and is regarded as the hepatic manifestation of the metabolic syndrome. Glucocorticoids can promote steatosis by stimulating lipolysis within adipose tissue, free fatty acid delivery to liver and hepatic *de novo* lipogenesis. Glucocorticoids can be reactivated in liver through 11β-hydroxysteroid dehydrogenase type 1 (11β-HSD1) enzyme activity. Inhibition of 11β-HSD1 has been suggested as a potential treatment for NAFLD. To test this, male mice with global (11β-HSD1 knockout [KO]) and liver-specific (LKO) 11β-HSD1 loss of function were fed the American Lifestyle Induced Obesity Syndrome (ALIOS) diet, known to recapitulate the spectrum of NAFLD, and metabolic and liver phenotypes assessed. Body weight, muscle and adipose tissue masses, and parameters of glucose homeostasis showed that 11β-HSD1KO and LKO mice were not protected from systemic metabolic disease. Evaluation of hepatic histology, triglyceride content, and blinded NAFLD activity score assessment indicated that levels of steatosis were similar between 11β-HSD1KO, LKO, and control mice. Unexpectedly, histological analysis revealed significantly increased levels of immune foci present in livers of 11β-HSD1KO but not LKO or control mice, suggestive of a transition to NASH. This was endorsed by elevated hepatic expression of key immune cell and inflammatory markers. These data indicate that 11β-HSD1-deficient mice are not protected from metabolic disease or hepatosteatosis in the face of a NAFLD-inducing diet. However, global deficiency of 11β-HSD1 did increase markers of hepatic inflammation and suggests a critical role for 11β-HSD1 in restraining the transition to NASH.

Nonalcoholic fatty liver disease (NAFLD) defines a spectrum of diseases ranging from simple steatosis to nonalcoholic steatohepatitis (NASH), fibrosis, progressing in rare cases to cirrhosis and hepatocellular carcinoma (1–3). Around 30% of the United States adult population has NAFLD with 3%–5% diagnosed with NASH (4), leading to increased morbidity and mortality (5).

Accumulating evidence supports an association between NAFLD and metabolic syndrome (MetS). Around 75% of obese people have NAFLD, with insulin resistance a key mechanistic factor between both conditions (6). As such, NAFLD can be regarded as the hepatic manifestation of the MetS, which together increase the risk of developing cardiovascular disease (6, 7).

Patients affected by glucocorticoid (GC) excess (Cushing syndrome) present with many of the disorders associated with MetS and can develop NAFLD (8). GCs promote steatosis through multiple mechanisms including stimulation of lipolysis within adipose tissue resulting in increased free fatty acid (FFA) delivery for utilization in the liver to produce lipids through enhanced hepatic de novo lipogenesis (9–11). In most patients with NAFLD and MetS, circulating GC concentrations are not elevated (12). However, GCs can be activated in a tissue-specific manner through the prereceptor activity of the 11β-hydroxysteroid dehydrogenase type 1 (11β-HSD1) enzyme, with the site of greatest activity being the liver (13). Thus, hepatic metabolism affected by GCs is a balance between circulating delivery and 11β-HSD1-mediated intracellular activation.

Preclinical studies using 11β-HSD1 knockout (KO) and transgenic mice have exemplified the role 11β-HSD1 can play in determining hepatic metabolic phenotype. Global deletion of 11β-HSD1 protects against high-fat diet induced obesity and glucose intolerance, whereas liver-specific 11β-HSD1 deletion does not (14–16). 11β-HSD1KO mice are protected from hepatosteatosis in the face of circulating GC excess and reveal the importance of adipose tissue in determining hepatic phenotype (14). Furthermore, mice with transgenic overexpression of 11β-HSD1, specifically in adipose and liver, develop hepatosteatosis in the context of a high-fat diet (17, 18).

Data from studies in humans support the idea that with steatosis there is decreased hepatic 11β-HSD1-mediated GC reactivation, possibly due to decrease local GC availability and preservation of metabolic phenotype (19). 11β-HSD1 inhibitors have been the subject of interest regarding their use in the treatment of conditions associated with MetS, with in-excess of 170 compounds having been developed for this purpose (20–23). Human clinical studies evaluating NAFLD patients showed that pharmacological inhibition of 11β-HSD1 was able to modestly reduce liver fat content over a 12-week treatment period, although whether this was a direct or peripheral effect was unclear (24).

Given the important role that 11β-HSD1-mediated GC metabolism plays in determining systemic and liver metabolic phenotype, we hypothesized that global deletion (11β-HSD1KO) and hepatocyte-specific deletion (LKO) mice would be protected from MetS and hepatosteatosis when subjected to a potent steatogenic diet. To this end, we fed 11β-HSD1KO mice the American Lifestyle Induced Obesity Syndrome (ALIOS) diet which, unlike a standard high-fat diet, is known to more faithfully recapitulate the spectrum of NAFLD, from steatosis to NASH (25).

Our data suggest that 11β-HSD1 loss of function affords no protection from ALIOS induced obesity, glucose intolerance, insulin resistance or hepatosteatosis. Unexpectedly, we show that in the context of hepatosteatosis, 11β-HSD1KO mice have increased inflammation, a prerequisite for the progression to NASH, as revealed by an accumulation of hepatic immune foci and increased expression of immune and inflammatory markers. These results suggest a role for 11β-HSD1 in restraining hepatic inflammation in NAFLD.

## Materials and Methods

### Animal husbandry

Male mice aged 7–8 weeks, with global (11β-HSD1KO) and hepatocyte-specific (LKO) deletions of 11β-HSD1 (16, 26); along with age-matched C57BL/6 control mice (Charles River) were used. Male mice were used as we did not want to deviate from the original ALIOS protocol, where advanced hepatosteatosis was attained using the feeding protocol described below (25). Mice were housed 2–3 per cage and maintained at the Biomedical Services Unit at the University of Birmingham, and all procedures conducted in accordance with the Animals (Scientific Procedures) Act 1986; regulated by the United Kingdom Home Office. Mice were maintained on a 12-hour light, 12-hour dark cycle at 21°C–22°C and were fed ad libitum, a diet consisting of 45% of calories from fat, of which 11.6% were derived from transfats (D13022701; Research Diets, Inc). Mice were also given a high fructose corn syrup equivalent drinking water replacement (55% fructose:45% glucose [wt/vol] deionized H_2_O at a concentration of 42 g/L). This feeding regimen, referred to as the ALIOS diet (25), was maintained for 16 weeks, after which mice were subjected to exsanguination via cardiac puncture under general anesthetic (isoflurane), followed by cervical dislocation; upon which tissues were excised and weighed. Once weighed, tissues were dissected then promptly snap-frozen in liquid nitrogen (LN_2_) or fixed in 4% formaldehyde (10% formalin [vol/vol] PBS).

### Metabolic analyses and plasma analytes

After 13 weeks of ALIOS treatment, blood from each mouse was collected into Microvette EDTA lined hematological tubes, from lateral tail veins and blood-glucose concentrations measured using an Accu-Chek monitor. The week after, mice were fasted overnight and glucose tolerance tests (GTTs) performed; whereby ip injections of 20% glucose (vol/vol) 0.9% sterile saline solutions at a dose of 2 g/kg were administered. Blood-glucose concentrations were measured at times (T)0, T15, T30, T60, T90, and T120 minutes after glucose injections. Blood samples were also collected at T0 and T30 after glucose injection and plasma insulin concentrations measured using the Ultra Sensitive Mouse Insulin ELISA kit (Crystal Chem).

Insulin tolerance testing (ITT) was performed during week 15 of the study. Mice were fasted for 4 hours and blood-glucose concentrations measured (T0), followed by the administration of insulin (Actrapid) via ip injection at a concentration of 0.1 IU/mL (0.1% 100 IU/mL [vol/vol] 0.9% sterile saline) at a dose of 0.75 IU/kg. Blood-glucose concentrations were subsequently measured at 15, 30, 60, 90, and 120 minutes after insulin injections.

Plasma FFA, triglyceride (TAG), and high-density lipoprotein (HDL)/low-density lipoprotein (LDL) cholesterol concentrations were measured from blood taken via terminal cardiac bleeds using protocols outlined according to the manufacturer's instructions (BioVision).

### Hepatic TAG quantification

Hepatic TAG content was ascertained using Biovision's colorimetric assay (Cambridge Bioscience). Briefly, 100 mg of frozen liver tissue was homogenized in 5% Nonidet P-40 before being heated for 5 minutes at 95°C in a water bath to solubilize TAGs, samples were left to cool to room temperature and the process repeated before centrifugation at 13 000 rpm. The resultant supernatants were diluted in distilled H_2_O and analyzed on a 96-well Wallac plate reader at λ570 nm.

### Histological analyses

Freshly excised livers were dissected, processed and embedded in paraffin wax, from which 5-μm sections were cut for analyses via histological staining. Hematoxylin and eosin (H&E) staining was used to score hepatosteatosis and inflammation using the NAFLD activity score (NAS) (27) and trichrome staining to access fibrosis. All images pertaining to histological analyses were viewed via light microscopy and photomicrographs taken using a Leica imaging system.

### Real-time quantitative PCR

Fragments of frozen liver tissue were homogenized using TRI Reagent (Sigma-Aldrich) for total RNA isolation. One microgram of total RNA was reversed transcribed to cDNA using Applied Biosystems High Capacity cDNA Reverse Transcription kit (Life Technologies) following the manufacturer's instructions. Reaction mixes consisting of Applied Biosystems TaqMan Universal PCR Master Mix and primer/probes (assays on demand), along with 1-μL cDNA, were made to 20 μL with nuclease-free water. All primer/probes targeted to genes of interest were labeled with FAM, whereas the reference gene, invariably 18s, was labeled with VIC; all reactions were singleplex and performed using Applied Biosystems ABI 7500 sequence detection system. Gene expression data was graphically represented as fold changes against controls. Reference genes' (18s) cycle threshold (Ct) values from each sample were subtracted from Ct values of corresponding samples genes of interest for δCt values (ΔCt). These values were then averaged and controls averages taken from those of KOs to give ΔΔCt values which were then incorporated into the equation 2^∧^ − ΔΔCt; to normalize cohort controls values to 1 and KO cohort as fold changes compared with controls (28).

### Statistical analyses

All data are derived from n = 13 controls, n = 11 11β-HSD1KO, n = 9 LKO, and n = 9 LKO control male mice with data presented as the mean ± SEM. Statistical analyses were performed using Mann-Whitney and Student's *t* tests and statistical significance assigned where *P* < .05. Although graphical representation of RT-qPCR data are presented as fold change verses controls, statistical analyses were performed using ΔCt values.

## Results

### Effect of ALIOS diet feeding on metabolic parameters

**Figure 1.**
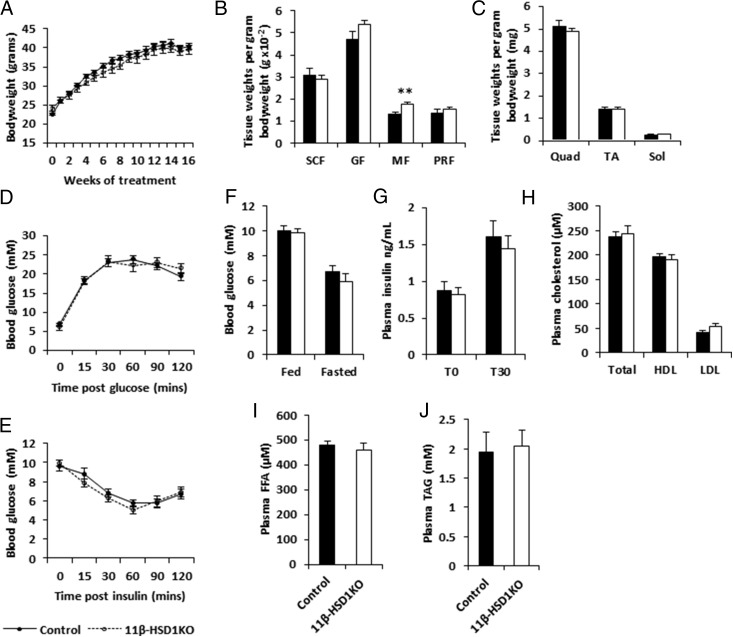
Loss of 11β-HSD1 does not protect mice from the adverse metabolic effects from being fed the ALIOS diet for 16 weeks. A, Body weight. B, Adipose depot weights; 11β-HSD1KO mice showed statistically significant differences only in mesenteric depot weights. C, Muscle bed weights. D, Glucose tolerance. E, Insulin tolerance. F, Fed and fasted blood glucose concentrations. G, Plasma insulin concentrations at T0 and T30 after glucose bolus injection. H, Total, HDL, and LDL plasma cholesterol concentrations. I, Plasma FFA. J, Plasma TAG. SCF, sc fat; GF, gonadal fat; MF, mesenteric fat; PRF, perirenal fat; TA, tibialis anterior. Mean ± SEM; **, *P* < .01 using Student's *t* test; n = 13 (controls) and n = 11 (11β-HSD1KO).

NAFLD is considered the hepatic manifestation of the MetS. Thus, we used the ALIOS diet as a means to induce MetS and fatty liver disease in 11β-HSD1KO and control mice. After 16 weeks of ALIOS diet, controls and 11β-HSD1KO mice showed similar rates of body weight gain (Figure 1A). Body weight normalized sc fat, gonadal fat, and perirenal fat pad weights showed no differences, with evidence for moderately increased accumulation of mesenteric fat in 11β-HSD1KOs compared with controls (*P* < .01) (Figure 1B). No differences were seen in lean mass (quadriceps, tibialis anterior, and soleus muscles) between 11β-HSD1KO and control mice (Figure 1C). 11β-HSD1KO mice showed no difference in glucose or insulin tolerance when compared with control mice (Figure 1, D and E). Furthermore, neither fed nor fasted state blood-glucose concentrations were different between 11β-HSD1KO and control mice (Figure 1F). Blood-insulin concentrations in the fasted state and 30 minutes after glucose bolus injection showed no differences between 11β-HSD1KO and control mice (Figure 1G). We also assessed plasma lipid profiles and show HDL and LDL cholesterol, FFAs and TAGs were no different between 11β-HSD1KO and control mice (Figure 1, H–J). Taken together, these data suggest that global deletion of 11β-HSD1 does not protect form the metabolic dysregulation associated with 16 weeks of ALIOS diet feeding, a time frame validated to cause obesity, insulin resistance, and dyslipidemia (25).

### Hepatic histology and steatosis scoring

**Figure 2.**
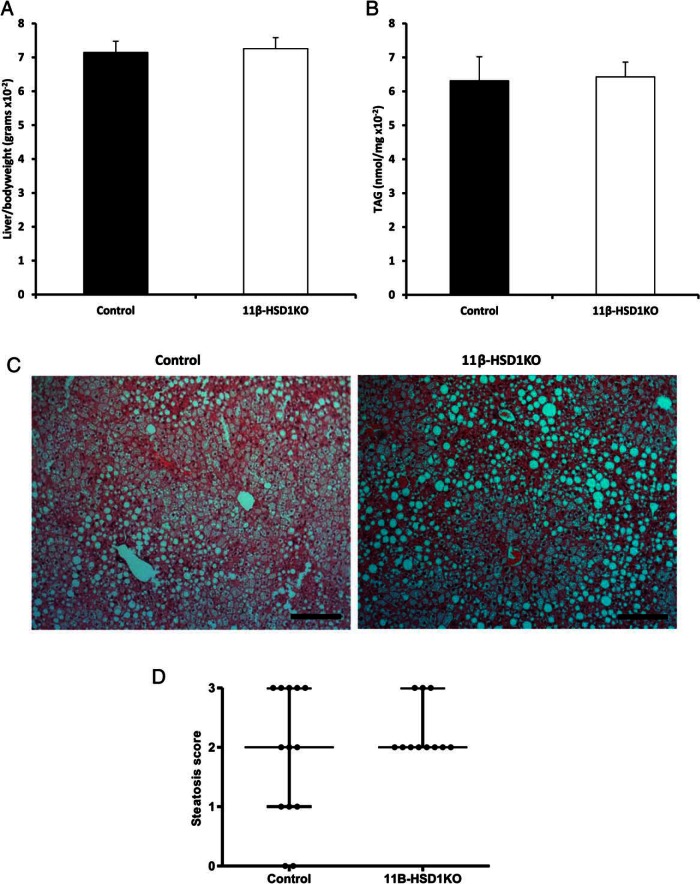
Loss of 11β-HSD1 does not reduce or delay hepatic lipid accumulation in mice fed the ALIOS diet for 16 weeks. A, Liver to body weight ratios in controls and 11β-HSD1KO. B, Hepatic TAG content in controls and 11β-HSD1KO mice. C, H&E-stained sections cut at 5 μm of controls and 11β-HSD1KO liver demonstrating levels of macro- and microvesicular steatosis. D, Dot plot representing steatosis in livers of control and 11β-HSD1KO mice was assessed using the NAS grading system. Mean ± SEM (A and B), interquartile range (D); n = 13 (control) and n = 11 (11β-HSD1KO). Scale bars, 200 μm.

ALIOS diet is also an established means to induce the classical features of hepatosteatosis and fatty liver disease (25), from which 11β-HSD1KO mice should be protected. However, there were no differences in the macroscopic appearance of the liver, or body weight normalized liver weight between 11β-HSD1KO and control mice (Figure 2A). Hepatic TAG content endorsed the liver weight data, with no differences observed (Figure 2B). Steatosis analysis, using blind scoring of H&E-stained livers of 11β-HSD1KO and control mice, showed microvesicular and macrovesicular steatosis in both 11β-HSD1KO and control mice. Although the severity varied across the cohorts, no differences in overall distribution in lipid accumulation were observed (Figure 2C). Indeed, qualitative assessment using NAS showed there was no statistically significant difference between hepatosteatosis in 11β-HSD1KO and control mice (Figure 2D). Overall, we find that deletion of 11β-HSD1 in mice treated with the ALIOS diet affords no protection from hepatic TAG accumulation or classical histological profile associated with hepatosteatosis and an overall MetS.

### Metabolic and hepatic analysis of hepatocyte-specific 11β-HSD1 deletion

**Figure 3.**
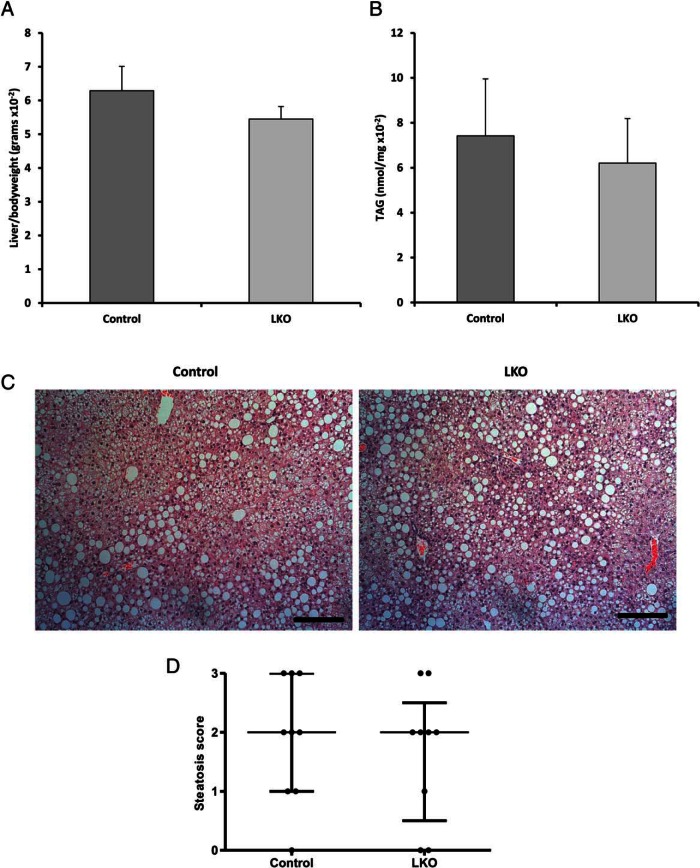
Mice with hepatocyte-specific deletion of 11β-HSD1 have steatosis comparable with global KO mice. A, Liver to body weight ratios in controls and LKO. B, Hepatic TAG content in controls and LKO mice. C, H&E-stained sections cut at 5 μm of control and LKO liver demonstrating levels of macro- and microvesicular steatosis. D, Dot plot representing steatosis in livers of control and LKO mice, assessed using the NAS grading system. Mean ± SEM (A and B), interquartile range (D); n = 9 (control) and n = 9 (LKO). Scale bars, 200 μm.

We also wished to delineate the contribution of hepatocyte specific-11β-HSD1 deletion to the development of a MetS and fatty liver in ALIOS-fed mice given the high level of enzyme expression in this cell type and its prominent role in GC-regulated lipid metabolism. To do this, we subjected hepatocyte-specific KO mice (LKO) to ALIOS diet for 16 weeks. As in 11β-HSD1KO, LKO mice gained weight at a similar rate to controls over the 16 weeks, with no discernible differences in end-point weight observed (control 34.73 ± 1.47 vs LKO 36.56 ± 0.70 g). No differences in body weight normalized adipose or lean tissue weights tissue weights were seen. In terms of glucose metabolism, area under the curve (AUC) analysis of GTTs and ITTs again showed no differences between LKO and control mice (GTT AUC, control 17.23 ± 2.26mM vs LKO 20.32 ± 2.22mM; ITT AUC, control 7.92 ± 0.33mM vs LKO 7.35 ± 0.37mM). Furthermore, there were no differences between normalized liver weight and hepatic TAG content between control and LKO mice, being equivalent to the 11β-HSD1KO (Figure 3, A and B). Hepatosteatosis analysis was conducted and showed equally distributed microvesicular and macrovesicular steatosis in both LKO and control mice (Figure 3C). NAS-assessed LKO and control livers showed similar levels of hepatosteatosis (Figure 3D). These data confirmed the overall view that ALIOS diet and its associated induction of MetS and NALFD is unaffected by the 11β-HSD1 loss of function.

### Hepatic inflammation analysis in 11β-HSD1KO mice

**Figure 4.**
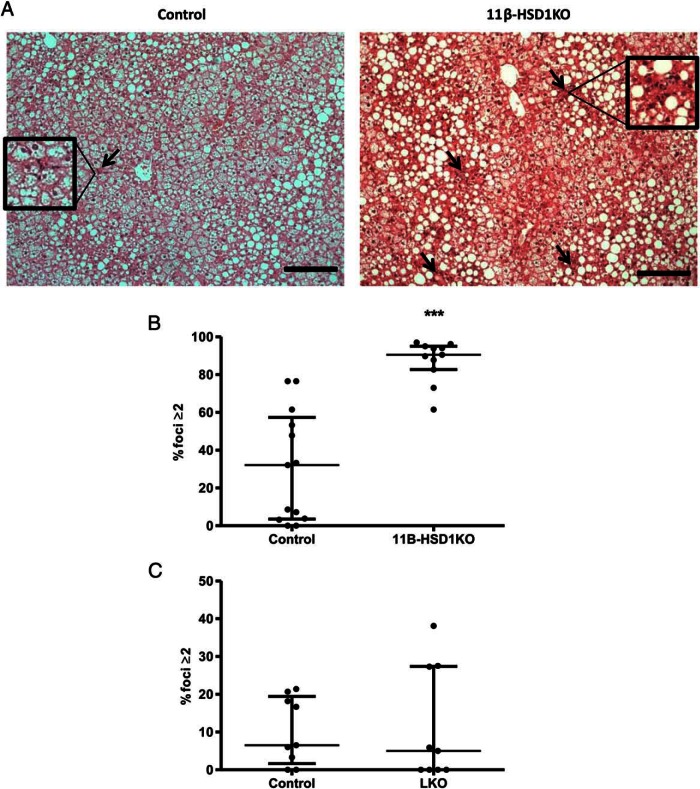
Loss of 11β-HSD1 results in an increased frequency of hepatic inflammatory foci compared with control after 16 weeks of ALIOS diet. A, H&E-stained sections cut at 5 μm show inflammatory foci (arrows) in livers of controls and 11β-HSD1KO mice, areas inside boxes highlight inflammatory foci (magnified an additional ×2). B, Dot plot showing % fields of view with 2 or more inflammatory foci; using the Mann-Whitney test, there was a significant increase in 11β-HSD1KO mice compared with controls, which was not seen in livers of LKO mice when compared with their controls (C). ***, *P* < .001, interquartile range; n = 13 (control), n = 11 (11β-HSD1KO), n = 9 (LKO control), and n = 9 (LKO). Foci per field of view at ×200 magnification. Scale bars, 200 μm.

It became apparent during the process of blind assessing histological NASs that certain sections displayed accumulations of hepatic immune foci in the context of steatosis. To better characterize this, blinded NAS system quantification of inflammatory and immune foci was conducted and revealed an average of 80% of fields of view from 11β-HSD1KO livers had 2 or more inflammatory foci compared with 30% in controls (*P* < .001) (Figure 4, A and B). Importantly, scoring of LKO immune foci showed no discernible differences with their controls (Figure 4C). These data suggest that despite no differences in systemic or hepatic metabolic parameters being observed, 11β-HSD1KO livers are potentially engaged in the early transition to inflammatory disease, and that extrahepatocyte 11β-HSD1 activity is important to this process.

### Markers of inflammation, immune cell infiltration, and fibrosis in 11β-HSD1KO mice

**Figure 5.**
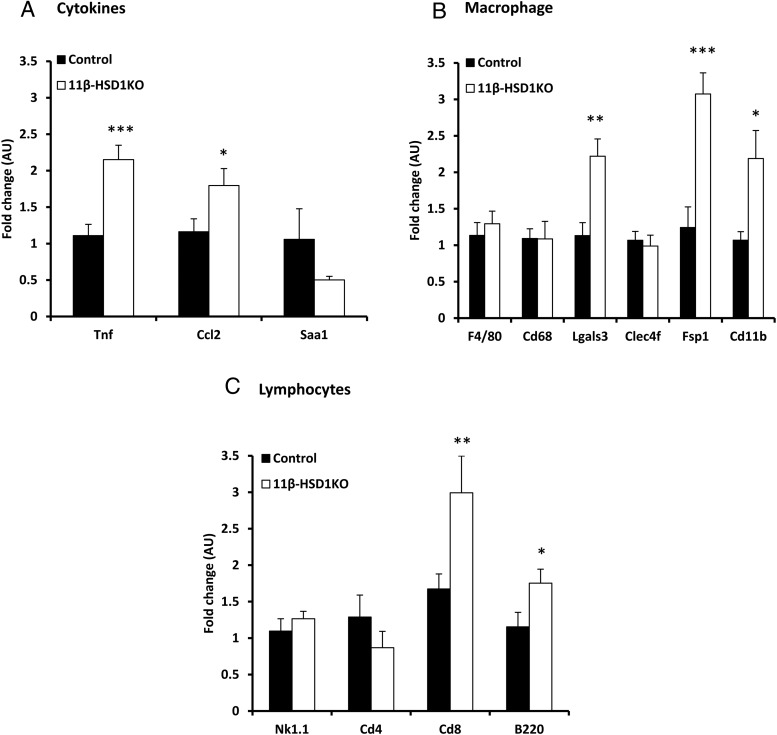
Increased hepatic expression of proinflammatory cytokines and macrophage markers in 11β-HSD1KO mice. A, Increased expression of proinflammatory cytokines *Tnf* and *Ccl2* in the livers of 11β-HSD1KO mice. B, Increased expression of macrophage specific markers, *Lgals3*, *Fsp1*, and *Cd11b* in 11β-HSD1KO compared with controls. C, Significant increases were seen in the expression of lymphocyte specific markers *Cd8* and *B220*. Mean ± SEM; *, *P* < .05; **, *P* < .01; ***, *P* < .001 using Student's *t* test; n = 13 (control) and n = 11 (11β-HSD1KO).

Following the observation of increased immune foci and early stage inflammatory disease in 11β-HSD1KO mice, we conducted gene expression analysis for markers of cytokines, macrophages, lymphocytes and fibrosis to further characterize this finding. The proinflammatory genes *Tnf* and *Ccl2* were significantly increased in livers of 11β-HSD1KO compared with controls (Figure 5A). Further to this, macrophage markers *Lgals3*, *Fsp1*, and *Cd11b*, known to be involved in the early modulation of NASH, were significantly increased, as were lymphocyte specific markers *Cd8* and *B220* in 11β-HSD1KO compared with controls (Figure 5, B and C). These suggest that 11β-HSD1 expression in nonhepatocyte cells is important to restraining the immune response to ectopic liver fat accumulation.

**Figure 6.**
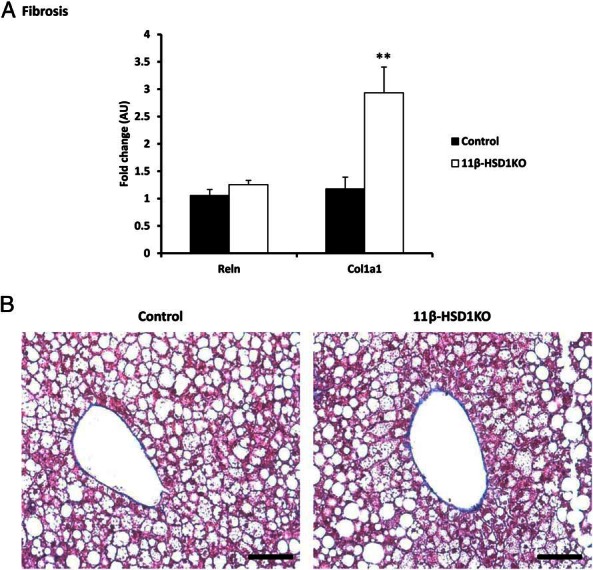
Increased expression of fibrosis-associated gene does not correlate with histological phenotype. A, Significant increases in *Col1a1* mRNA, a marker of fibrosis, in 11β-HSD1KO mice. Hepatic stellate cell marker Reelin (*Reln*), was not increased. B, Assessment of fibrosis using trichrome stained sections cut at 5 μm showed no ectopic collagen in either control or 11β-HSD1KO mice. Mean ± SEM; **, *P* < .01 using Student's *t* test; n = 13 (control) and n = 11 (11β-HSD1KO). Scale bars, 100 μm.

Because the pathogenesis of NASH involves the deposition of collagen and the formation of fibrotic lesions as hallmark features of advancing disease, we assessed the expression of fibrosis markers and show that *Col1a1*, encoding the major protein component of type 1 collagen, is significantly increased (*P* = .004) in 11β-HSD1KO but not in control livers. Reelin (*Reln*) expressed by hepatic stellate cells can act as a marker of hepatocyte damage, and was shown not to be altered (Figure 6A). However, trichrome staining showed no ectopic collagen deposition evident in either 11β-HSD1KO or control liver, with only collagen of the tunica externa of blood vessels stained (Figure 6B), suggesting that increased *Col1a1* expression was a marker of the very earliest progression to NASH only seen in 11β-HSD1KO mice.

## Discussion

Mice deficient in 11β-HSD1, or treated with 11β-HSD1 inhibitors, resist obesity and a MetS phenotype when fed conventional high-fat diets (15, 21). Based on observations from such models and recent human clinical studies we postulated that 11β-HSD1KO and hepatocyte-specific KO (LKO) mice would be protected against the development of MetS and resist hepatosteatosis when challenged with the ALIOS diet. After 16 weeks of ALIOS, we show that male 11β-HSD1KO and LKO mice are indistinguishable from control mice displaying obesity, glucose intolerance, dyslipidemia and indeed hepatosteatosis. An increase in mesenteric adipose tissue mass was measured in 11β-HSD1KO mice. This runs contrary to that found for 11β-HSD1KO mice fed a high-fat diet, in which there was preferential adipose deposition away from the mesenteric depot (15). Nonetheless, positive correlations have been shown between mesenteric fat mass and hepatosteatosis in human subjects (28–30).

Unlike conventional high-fat diets, ALIOS diet includes transfats and fructose, both of which can drive hepatic lipid metabolism and accumulation, and have been shown to exacerbate the development of MetS beyond that achieved by conventional high-fat diets (25, 31–33). Indeed ALIOS is also validated to induce a more vigorous progression from simple steatosis to NASH, something that high-fat diets rarely achieve (25, 34).

We had further hypothesized that KO mice would be protected from developing overt fatty liver and show a decrement in lipid accumulation. However, hepatic lipid accumulation and quantitative NAS analysis demonstrated no differences between 11β-HSD1KO, LKO, and their control mice. A number of preclinical animal models have demonstrated the potential for 11β-HSD1 to modulate lipid accumulation and the NAFLD phenotype. Mice with transgenic over expression of 11β-HSD1, specifically in adipose and liver, develop steatosis in the context of a HFD (17, 18). 11β-HSD1KO and adipose-specific 11β-HSD1KO mice resist hepatosteatosis when GCs are in circulatory excess, achieved through the lack of adipose 11β-HSD1 limiting lipolysis and hepatic FFA delivery, whereas LKO maintained florid steatosis as in wild-type mice (14, 16). Furthermore, a recent phase 1B clinical trial using the 11β-HSD1 inhibitor RO5093151 effectively reduced liver-fat content of those treated; however, the tissues mediating this effect were not identified (24).

On this basis, we conclude that in the context of the ALIOS diet, neither global nor hepatocyte-specific 11β-HSD1 loss of function in male mice has any protective effect against the development of hepatosteatosis.

Simple and benign steatosis is a reversible condition which precedes more destructive NASH, fibrosis and cirrhosis (35). Our unanticipated observations of inflammatory disease only, in 11β-HSD1KO mice, implied that we had collected liver transitioning to the early stage of NASH. Inflammatory scoring was not a primary end-point measure in terms of our initial hypothesis regarding metabolic disease. However, there is accumulating evidence for a prominent role for 11β-HSD1 in modulating local tissue inflammatory responses, regulating early restraint of the acute inflammatory process. 11β-HSD1 deficiency has been shown to worsen acute inflammation with greater infiltration of inflammatory cells into target sites in experimental models of rheumatoid arthritis and myocardial infarction (36, 37). However, this is not a universal observation. Reduced inflammation in adipose tissue has been observed in 11β-HSD1KO mice, suggesting tissue-specific inflammatory phenotypes are important (38).

We endorsed our histological observations by showing elevated expression of markers of inflammation and immune cell infiltration/activation in livers of 11β-HSD1KO mice when compared with controls. Fibroblast-specific protein-1 (*Fsp1*/*S100a4*), a marker for a subpopulation of macrophages specific to liver damage was increased yet *F4/80* and *Cd68*, pan macrophage markers, and *Clec4f*, expressed in Kupffer and migrant liver macrophages were not. Although no firm conclusions can be drawn, they do suggest differential regulation of FSP1^+ve^ macrophages in the context of 11β-HSD1 and diet-induced hepatic inflammation. Importantly, FSP1^+ve^ macrophages also express *Cd11b*, *Ccl2*, and *Tnf*, all of which were also significantly increased in 11β-HSD1KO mice. Because several liver resident immune cells types express these markers, elucidation of the subpopulations involved could not be ascertained (39–42). In healthy livers, resident immune cells are found in portal tracts and migrate into the parenchyma upon initiation of an inflammatory response (43–45). It has been shown that they automodulate their physiology via intracrine 11β-HSD1-mediated reactivation of GC, regulating processes such as mast cell degranulation, dendritic cell differentiation, and neutrophil and dendritic cell susceptibility to apoptosis (46–50). This evidence suggests the intrinsic ability of immune cells to autoregulate availability of GC is crucial to appropriate immune function.

We postulate that ALIOS induced hepatic inflammation is initially restrained by extrahepatocyte 11β-HSD1 activity in resident hepatic immune cells or/and by peripheral extravasated immune cells such as macrophages (51, 52). This is supported by data showing macrophages deficient in 11β-HSD1 fail to phagocytose apoptotic neutrophils when treated in vitro with 11β-HSD1 substrate; with 11β-HSD1KO mice having delayed macrophage phagocytic competence during induced peritonitis (49). The enhanced inflammatory phenotype exhibited by 11β-HSD1KO mice also endorses clinical data, whereby steatohepatitis was accompanied with an increase in 11β-HSD1, suggesting a requirement for increased GC activity in inflamed livers (19).

Although we show significantly increased hepatic expression of *Col1a1*, an early marker of liver collagen deposition (53), we could not demonstrate histological evidence of fibrosis, and may be due to 16 weeks of ALIOS being insufficient to evoke fibrotic lesions. ALIOS diet fed for 12 months has been shown to recapitulate the development of NASH, as 80% of animals developed fibrosis and 60% hepatocellular carcinoma (34).

These data indicate ALIOS as a useful means to examine the relationship between hepatic 11β-HSD1-mediated GC metabolism, MetS and inflammatory disease. Importantly, we highlight the need to further explore 11β-HSD1 in the context of hepatic inflammation and its role in the mechanisms that gateway the development of NASH. Further work is required to elucidate the cell types and mechanisms critical to resolving hepatic inflammation in the setting of ALIOS induced NAFLD and MetS. Ultimately, the clinical application of 11β-HSD1 inhibitors as treatments for many of the conditions associated with MetS may not be advisable when NAFLD is also present.
